# Rescue from acute neuroinflammation by pharmacological chemokine-mediated deviation of leukocytes

**DOI:** 10.1186/1742-2094-9-243

**Published:** 2012-10-25

**Authors:** Nele Berghmans, Hubertine Heremans, Sandra Li, Erik Martens, Patrick Matthys, Lydia Sorokin, Jo Van Damme, Ghislain Opdenakker

**Affiliations:** 1 Rega Institute for Medical Research, Department of Microbiology and Immunology, University of Leuven, Leuven, Belgium; 2 Institute of Physiological Chemistry and Pathobiochemistry, Münster University, Münster, Germany

**Keywords:** Encephalitis, Leukocytes, Chemokines, Central nervous system

## Abstract

**Background:**

Neutrophil influx is an important sign of hyperacute neuroinflammation, whereas the entry of activated lymphocytes into the brain parenchyma is a hallmark of chronic inflammatory processes, as observed in multiple sclerosis (MS) and its animal models of experimental autoimmune encephalomyelitis (EAE). Clinically approved or experimental therapies for neuroinflammation act by blocking leukocyte penetration of the blood brain barrier. However, in view of unsatisfactory results and severe side effects, complementary therapies are needed. We have examined the effect of chlorite-oxidized oxyamylose (COAM), a potent antiviral polycarboxylic acid on EAE.

**Methods:**

EAE was induced in SJL/J mice by immunization with spinal cord homogenate (SCH) or in IFN-γ-deficient BALB/c (KO) mice with myelin oligodendrocyte glycoprotein peptide (MOG_35-55_). Mice were treated intraperitoneally (i.p.) with COAM or saline at different time points after immunization. Clinical disease and histopathology were compared between both groups. IFN expression was analyzed in COAM-treated MEF cell cultures and in sera and peritoneal fluids of COAM-treated animals by quantitative PCR, ELISA and a bioassay on L929 cells. Populations of immune cell subsets in the periphery and the central nervous system (CNS) were quantified at different stages of disease development by flow cytometry and differential cell count analysis. Expression levels of selected chemokine genes in the CNS were determined by quantitative PCR.

**Results:**

We discovered that COAM (2 mg i.p. per mouse on days 0 and 7) protects significantly against hyperacute SCH-induced EAE in SJL/J mice and MOG_35-55_-induced EAE in IFN-γ KO mice. COAM deviated leukocyte trafficking from the CNS into the periphery. In the CNS, COAM reduced four-fold the expression levels of the neutrophil CXC chemokines KC/CXCL1 and MIP-2/CXCL2. Whereas the effects of COAM on circulating blood and splenic leukocytes were limited, significant alterations were observed at the COAM injection site.

**Conclusions:**

These results demonstrate novel actions of COAM as an anti-inflammatory agent with beneficial effects on EAE through cell deviation. Sequestration of leukocytes in the non-CNS periphery or draining of leukocytes out of the CNS with the use of the chemokine system may thus complement existing treatment options for acute and chronic neuroinflammatory diseases.

## Background

Neuroinflammation is a common denominator in a wide variety of diseases of the central nervous system (CNS), ranging from various forms of acute infectious or vascular meningoencephalitis to chronic inflammation associated with multiple sclerosis (MS) or neurodegenerative diseases. Hyperacute experimental autoimmune encephalomyelitis (EAE), henceforth named as such to distinguish it from T cell-mediated forms of EAE, is induced by immunization of mice with spinal cord homogenates, has an important neutrophil component [1] and is an appropriate model for the study of neuroinflammation in acute encephalitis. By contrast, chronic forms of EAE are often induced with CNS peptide antigens or by adoptive transfer of neuroantigen-specific T cell clones or T cells from sensitized animals, and are widely used to study disease mechanisms and new therapeutic approaches for MS [2].

Various aspects of recent research point to a role of the blood–brain barrier (BBB) as a crucial structure to prevent neuroinflammation [3,4]. Neuroinflammation occurs upon migration of leukocytes through the endothelial and parenchymal layers of the BBB in order to gain access to the CNS parenchyma [5] and as a result of chemokine-governed attraction of specific leukocytes to the CNS [6]. Current ways to prevent leukocyte entry into the CNS include treatment with IFN-α/β or anti-adhesive agents that prevent binding of leukocytes to CNS endothelium [7,8].

IFN-β has been proven effective for the treatment of relapsing remitting MS [7]: IFN-β decreases the relapse rate, ameliorates disease activity and reduces the number of inflammatory CNS lesions [9]. Many studies have shown that IFN-β exerts beneficial effects on the development of EAE and on the stability of the BBB [10-12]. Therefore, induction of endogenous IFN-β is an alternative approach to substitution therapy with IFN-β. In early studies, such stimulation was typically achieved by induction of IFN-β with viral double-stranded RNAs or therapeutically by polyanions, including polyI:C and polyacrylates [13,14]. PolyI:C has been reported to suppress disease in a murine model of relapsing EAE by inducing endogenous IFN-β but has high toxicity [15]. Chlorite-oxidized oxyamylose (COAM), a synthetic polyanion with a therapeutic index of 300 to 500, is a potent antiviral agent [16], which we recently showed to result in almost complete protection against acute infection with the neurotropic mengovirus. Similarly, COAM has no cytotoxic effects *in vitro*[17,18]. In this animal model of infection, COAM was shown to induce myeloid cell chemotaxis, in part through binding and activity of the chemokine granulocyte chemotactic protein-2/CXCL6 [19]. In view of the supposed IFN-inducing capacity of COAM, we started a series of studies to determine its effects in EAE models. COAM was shown to suppress neuroinflammation significantly by interfering with the chemokine system and by causing retention of immune cells at the peritoneal injection site, thus affecting cell fluxes to the brain. These results suggest that diverting leukocyte chemotaxis from the brain into the non-CNS periphery may represent a novel and pharmacologically attainable treatment option that complements existing therapies for various forms of acute neuroinflammation.

## Methods

### Mice

SJL/J and IFN-γ KO mice were bred under conventional conditions in the Experimental Animal Breeding Facility of the University of Leuven, Belgium. The generation and basic characterization of IFN-γ-deficient mice of the 129 x BALB/c strain have been described previously [20]. These mice were backcrossed for eight generations to the parental BALB/c strain. EAE experiments were carried out with 8 to 10 week old male and female mice. During the experiments, mice were kept under conventional housing conditions. They received a regular diet and acidified drinking water without antibiotics. All procedures were conducted in accordance with protocols approved by the local Ethics Committee (Licence number LA1210243, Belgium).

### Reagents

*Mycobacterium tuberculosis* strain H37Ra, Incomplete Freund’s Adjuvant (IFA) and Complete Freund’s Adjuvant (CFA) were purchased from Difco Laboratories (Detroit, MI, USA). Pertussis toxin was purchased from List Biological Laboratories (Campbell, CA, USA). COAM was prepared as described [21]. It was free of endotoxin (<13.3 pg/mg COAM, assayed in the *Limulus* amoebocyte lysate assay) and devoid of contaminating proteins (assayed by protein staining) [18]. Myelin oligodendrocyte glycoprotein peptide (MOG_35-55_) was produced by Fmoc (fluorenylmethoxycarbonyl) solid phase peptide synthesis, purified by reversed phase chromatography and peptide mass was confirmed by electrospray ion trap mass spectrometry [22].

### Induction and clinical evaluation of EAE and treatment with COAM

For induction of hyperacute EAE in SJL/J mice, an emulsion was prepared consisting of 100 mg/ml of lyophilized SJL/J mouse spinal cord homogenate (SCH) in PBS and 4 mg/ml *M. tuberculosis* (strain H37Ra) in CFA. Chronic EAE was induced in IFN-γ KO BALB/c mice by injecting 50 μg of MOG_35-55_ peptide (1 mg/ml in saline) emulsified in IFA containing 4 mg/ml of *M. tuberculosis*. On day 0, mice were injected subcutaneously in each of the two hind footpads with 50 μl of the emulsion. Immediately thereafter and on day 2, 100 ng of pertussis toxin in 50 μl saline was intravenously (i.v.) administered in the tail vein. Animals were anaesthetized for injections.

Mice were evaluated daily for signs of clinical disease. Disease severity was recorded as follows: grade 0, normal; grade 0.5, floppy tail; grade 1, tail paralysis and mild impaired righting reflex; grade 2, mild hind limb weakness and impaired righting reflex; grade 3, moderate to severe hind limb paresis and/or mild forelimb weakness; grade 4, complete hind limb paralysis and/or moderate to severe forelimb weakness; grade 5, quadriplegia or moribund; grade 6, death.

COAM is hydrophilic and was dissolved in pyrogen-free 0.9% NaCl. Mice were treated with an intraperitoneal (i.p.) injection of COAM (2 mg in 0.2 ml 0.9% NaCl) on days 0 and/or 7 after EAE immunization. Control mice received an equivalent volume of saline (0.9% NaCl).

### Isolation and induction of mouse embryonic cells

Mouse embryonic fibroblasts (MEF) were isolated from SJL/J mouse embryos around 17 days of gestation. The uterine horns were dissected and placed in a petri dish containing MEM (Invitrogen, Paisly, Scotland) supplemented with penicillin (500 U/ml; Continental Pharma, Brussels, Belgium) and streptomycin (500 μg/ml; Continental Pharma). Each embryo was separated from its placenta and surrounding membranes and washed three times with MEM. Subsequent procedures were according to standard conditions: incubation of embryo fragments in 50 ml trypsin-ethylenediaminetetraacetic acid (EDTA), centrifugation of cell suspension at 135 g for 15 minutes, two washings of the cell pellets with MEM growth medium containing 10% heat-inactivated FCS, 200 mM L-glutamine and 0.1% sodium bicarbonate (Invitrogen) and culture of adherent cells to confluency in growth medium in flat-bottomed flasks (75 cm^2^, TPP, Zurich, Switzerland) for four days.

For the induction of IFN-β in MEF, 1 x 10^6^ cells in a total volume of 2 ml growth medium were seeded in six-well plates (TPP). After incubation for 24 hours, cells were stimulated with different concentrations of COAM in MEM with 2% FCS for 72 hours. Supernatants were collected for detection of IFN-β with a biological antiviral assay on IFN-sensitive fibroblastoid L929 cells and with ELISA for IFN-γ determination. The MEF cells were harvested after 72 hours and used for quantitative PCR (qPCR) analysis of cytokine and chemokine mRNAs.

### Relative quantitation of cytokine, chemokine and chemokine receptor mRNAs by qPCR

Total RNA was purified from cells or tissues (RNeasy Mini Kit, Qiagen, Venlo, The Netherlands) and transcribed into cDNA using the High Capacity cDNA Reverse Transcription Kit (Applied Biosystems, Foster City, CA, USA). qPCR reactions were carried out in an ABI Prism 7000 Sequence Detection System (Applied Biosystems) in a total volume of 30 μl, containing 50 ng of extracted RNA, 15 μl of TaqMan Gene Expression Master Mix (Applied Biosystems) and 1.5 μl of primer/probe mix for the appropriate cytokine, chemokine or chemokine receptor (TaqMan Gene Expression Inventoried Assays, Applied Biosystems). The qPCR conditions consisted of an initial step at 50°C for 2 minutes, an activation step at 95°C for 10 minutes followed by 40 cycles of 15 seconds at 95°C and 1 minute at 60°C. 18S or glyceraldehyde 3-phosphate dehydrogenase (GAPDH) (Applied Biosystems) were included as endogenous reference genes for normalization of target mRNA transcripts. The fold change in gene expression normalized to the endogenous reference and relative to the untreated control was determined according to the comparative ΔΔCt method [23].

### Bioassay on L929 cells for detection of IFN activity

IFN assays were carried out as described [24]. Briefly, mouse L929 cells were seeded in flat-bottom 96-well plates at a density of 6 x 10^4^ cells per well in MEM growth medium. In each assay a laboratory mouse IFN standard (consisting of mouse L929 cell-derived IFN-αβ, induced with Newcastle disease virus) was included and 0.5 log_10_ dilutions of the samples were made in growth medium. After 24 hours of incubation at 37°C, the cultures were challenged with 50 μl of mengovirus (multiplicity of infection, 0.01 plaque-forming units per cell). Cell controls received growth medium only. Plates were incubated at 37°C for 24 hours and cells were colored with crystal violet. The detection limit of this biological IFN assay was 3.16 units/ml.

### Detection of cytokines and chemokines by ELISA

Mouse IFN-γ concentrations were determined by the sandwich ELISA described previously [25]. Briefly, samples were incubated in microtiter plates coated with mouse anti-rat IFN-γ-specific mAb as capturing antibody (DB1; gift from Dr. P. van der Meide, Cytokine Biology Unit, Central Laboratory Animal Institute, Utrecht University, Utrecht, The Netherlands). The bound cytokine was detected by incubation in turn with rat anti-mouse IFN-γ-specific mAb (F1), used as primary detection antibody, and goat anti-rat immunoglobulin-peroxidase conjugate (Jackson ImmunoResearch Laboratories, West Grove, PA, USA) as secondary detection antibody.

IFN-β protein concentrations in sera of IFN-γ KO mice were quantified using the VeriKine Mouse Interferon Beta ELISA kit (PBL InterferonSource, Piscataway, NJ, USA). GCP-2 was detected by an ELISA developed in our laboratory, as described [26]. IL-17, KC and MIP-2 levels were measured by sandwich ELISA using paired antibodies according to the manufacturer’s recommendations (DuoSet ELISA Development System, R&D Systems, Abingdon, UK).

### Cell preparation from various organs

#### Brain and spinal cord

Mice were sacrificed and gently perfused through the left cardiac ventricle with 50 ml ice-cold PBS to eliminate intravascular contaminating blood cells in the CNS. Spinal cords were removed by flushing the spinal column with sterile PBS and brains were dissected. Both tissues were homogenized and filtered through a cell strainer (Becton Dickinson Labware, Franklin Lakes, NJ, USA). After centrifugation (10 minutes, 300 g), the cells were resuspended in 40% Percoll and underlayed with 72% Percoll. The gradient was centrifuged at 500 g for 20 minutes at 10°C. Before staining analysis, the interphase cells were collected and extensively washed in PBS supplemented with 2% FCS.

#### Blood

Peripheral blood samples were taken at the orbital sinus, using heparin as an anticoagulant. Leukocytes were obtained by lysis of red blood cells by two incubations (5 and 3 minutes, 37°C) in NH_4_Cl solution (0.83% w/v in 0.01M Tris/HCl; pH 7.2). Remaining cells were washed two times with ice-cold PBS, supplemented with 2% FCS, and then analyzed.

#### Spleen

Spleens were isolated, cut into small pieces and passed through cell strainers, to obtain single cell suspensions. Red blood cells were lysed by two incubations (5 and 3 minutes at 37°C) of the splenocyte suspension in NH_4_Cl solution (0.83% w/v in 0.01M Tris/HCl; pH 7.2). Remaining cells were washed two times with ice-cold PBS containing 2% FCS.

#### Peritoneal fluid

Peritoneal lavage fluid was collected following killing of the mice. Five ml of ice-cold PBS containing 2% FCS was injected i.p. and the abdominal space was gently massaged. The lavage fluid was collected and centrifuged at 300 g for 10 minutes.

### Flow cytometry analysis

Single cell suspensions (0.5 x 10^6^ cells) were incubated for 15 minutes with the Fc-receptor-blocking antibodies anti-CD16/anti-CD32 (BD Biosciences Pharmingen, San Diego, CA, USA), washed with PBS supplemented with 2% FCS and then stained for 30 minutes with the indicated fluorescein isothiocyanate (FITC)-conjugated and phycoerythrin (PE)-conjugated antibodies. Cells were washed twice and fixed with 0.37% formaldehyde in PBS. FITC-conjugated anti-CD8, PE-conjugated anti-CD4, FITC-conjugated anti-CD11b, PE-conjugated anti-Gr-1 and PE-conjugated anti-F4/80 were purchased from eBioscience (San Diego, CA, USA). Cells were analyzed by a FACSCalibur flow cytometer and data were processed with the CellQuest software (Becton Dickinson).

### Differential cell counting and histopathology analysis

Cells from spinal cord and brain and from other anatomical compartments were applied to slides by centrifugation at a density of approximately 10^5^ cells/slide using a Shandon Cytospin 2. Cytospin preparations were stained with Hemacolor (Merck, Darmstadt, Germany) and leukocytes were identified on the basis of morphology. Five series of 100 cells from each slide were counted and the results were expressed as a percentage of the total cell count.

Spinal cords and brains were fixed in 4% formalin. Four micron thick paraffin sections were stained with H & E and scored for signs of neuroinflammation by two independent observers.

### Statistical analyses

Differences in the clinical course of EAE were analyzed by Wilcoxon’s non-parametric test or, where appropriate, by the Chi-square test with Yates’ correction. Significant differences between groups were evaluated using a non-parametric Mann Whitney test. All *P* values of 0.05 or less were considered significant.

## Results

### COAM protects against hyperacute and chronic EAE without inducing interferon-β

**Figure 1:**
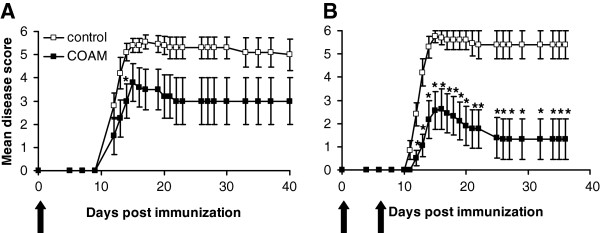
**The clinical course of hyperacute EAE in COAM-treated mice.** (**A**) SJL/J mice were immunized subcutaneously with syngeneic SCH for induction of hyperacute EAE and treated i.p. at day 0 with 2 mg COAM. Mean disease scores were calculated for COAM-treated (n = 10, filled squares) or saline-treated (n = 10, open squares) animals. One significant data point is indicated by an asterisk at day 14. (**B**) When two injections with COAM were given on days 0 and 7 after immunization, significant reduction of disease scores (asterisks) were observed from day 12 onwards. The clinical course of hyperacute EAE in these mice is shown as mean daily group score ± SEM. Dead animals were scored grade 6 from the day of death until the end of the study. Black vertical arrows indicate time points of COAM administration. * *P* <0.05 for comparison with saline-treated group (Wilcoxon test). COAM, chlorite-oxidized oxyamylose; EAE, experimental autoimmune encephalomyelitis; SCH, spinal cord homogenate; SEM, standard error of the mean.

Monophasic hyperacute EAE in SJL/J mice was induced by immunization with syngeneic SCH in CFA and mice were treated by injection of COAM (2 mg i.p.) at various time points. Control mice consisted of EAE-induced animals treated with excipiens and untreated naive mice were used to measure background levels of all parameters. The first signs of hyperacute EAE appeared between days 11 and 13 after immunization. Mice treated with a single dose of COAM on the day of immunization (day 0) had significantly less severe clinical signs at day 14 (*P* < 0.05; indicated by single asterisk in Figure 1, panel A) and decreased incidence of the disease to 50% (*P* <0.05) (Figure 1A, Table 1) compared to saline-treated control animals. These results indicate that a single injection of COAM results in effects that last several days. By contrast, a single i.p. injection of COAM on day 8 after immunization was ineffective in this animal model (Table 1). Mice treated with COAM on days 0 and 7 after immunization exhibited a stronger reduction of hyperacute EAE compared to those treated with a single dose of COAM at day 0 (Figure 1A and 1B), with significantly reduced severity of clinical signs and mortality rates (*P* <0.05 and *P* <0.01, respectively) compared to saline–treated mice (90% mortality) (Table 1). These *in vivo* results indicated that two i.p. injections of COAM at days 0 and 7 after immunization provided partial protection against SCH-induced hyperacute EAE and this significantly decreased the mean daily disease score from the onset of clinical signs until the termination of the experiment at day 40. This effect was dose-dependent (with decreased survival rates after treatment with 0.5 mg per mouse).

**Table 1:** Hyperacute EAE development in SJL/J mice treated with COAM

	**Day 0**		**Day 8**		**Day 0 + 7**	
	**saline**	**COAM**	**saline**	**COAM**	**saline**	**COAM**
Mean maximum disease score ± SEM	5.6 ± 0.25	3.8 ± 0.78*	3.6 ± 0.75	4.6 ± 0.62	5.75 ± 0.24	2.72 ± 0.79*
Incidence	10/10	5/10*	8/10	9/10	10/10	6/9
Mortality	8/10	5/10	4/10	6/10	9/10	2/9**
Mean day of onset ± SEM	12.3 ± 0.2	13.1 ± 0.37	13 ± 0.56	12.2 ± 0.14	11.8 ± 0.28	12.8 ± 0.37

SJL/J mice were immunized subcutaneously with syngeneic SCH for induction of EAE as described in Materials and Methods. They were treated i.p. with 2 mg COAM or saline on the indicated days after immunization. Dead animals were scored 6 from the day of death until the end of the study. * *P* <0.05; ** *P* <0.01 for comparison with saline-treated group; Wilcoxon test (disease score) or Chi-square test (incidence and mortality). COAM, chlorite-oxidized oxyamylose; EAE, experimental autoimmune encephalomyelitis; SCH, spinal cord homogenate; SEM, standard error of the mean.

**Figure 2:**
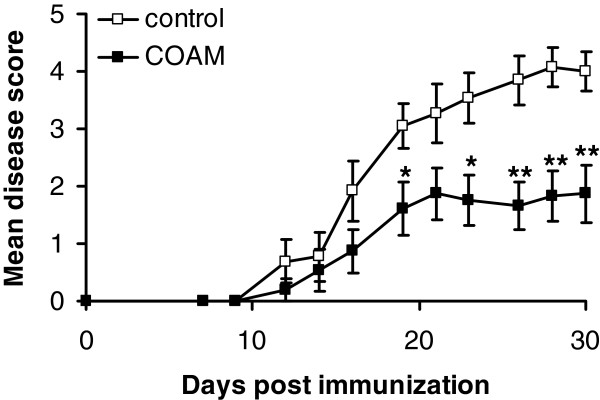
**COAM reduces MOG peptide-induced EAE.** IFN-γ KO BALB/c mice were immunized with MOG_35-55_ and the test group was given 2 mg COAM/mouse i.p. at days 0 and 7 after immunization, whereas the control group received excipiens. EAE clinical scores were recorded daily. Dead animals were scored 6 from the day of death until the end of the experiment. * *P* <0.05; ** *P* <0.01 for comparison with saline-treated group (Wilcoxon test, control n = 13, COAM n = 15). COAM, chlorite-oxidized oxyamylose; EAE, experimental autoimmune encephalomyelitis; MOG, myelin oligodendrocyte glycoprotein.

In order to validate these data in other EAE models, we tested COAM in antigen-specific EAE using immunization of IFN-γ KO BALB/c mice with MOG_35-55_ peptide and a treatment schedule with COAM on days 0 and 7. Figure 2 shows the cumulated results of three independent experiments. These demonstrated that COAM (mean maximal disease score: 2.37 ± 0.47; incidence: 11/15; mean day of disease onset: 17.55 ± 1.0) protected significantly against MOG peptide-induced EAE in comparison with control animals (mean maximal disease score: 4.27 ± 0.29; incidence: 13/13; mean day of onset: 17.31 ± 1.16).

After administration of COAM or other polyanions in viral infection models, low levels of IFN are detectable in the serum during the first 24 to 48 hours, with peak IFN levels after 18 hours [16]. However, more recent antiviral studies have suggested that COAM does not induce IFN-β *in vitro*[18] or *in vivo*[19]. Hence, it is not clear whether COAM can induce IFN or whether virus challenge is the main inducer of IFN. To distinguish between these possibilities it is necessary to examine whether COAM can induce IFN-β in virus-free conditions. Therefore, we tested extensively whether IFN-β which protects against EAE [11], is upregulated in COAM-treated hyperacute EAE animals or cell cultures.

First, groups of SJL/J mice induced for EAE were bled 6, 18 and 24 hours after each COAM administration (2 mg i.p. on days 0 and 7 after immunization). Sera were tested for IFN bio-activity on L929 cells with the use of a viral cytopathogenic reduction assay. This analysis revealed no IFN-like activity (detection limit <3.16 units/ml) in any of the test samples, whereas standard IFN-α/β preparations yielded the expected titers between 2.0 to 2.5 log_10_ units/ml. In addition, no IFN-γ was detectable by a specific ELISA in any of the titrated sera samples (detection level was 1.25 units/ml). Similarly, peritoneal fluids from saline- and COAM-treated EAE mice, collected at days 5, 9 and 16 after immunization, did not contain any IFN-like activity. Sera from EAE-induced IFN-γ KO mice (collected at 0, 6, 18 and 24 hours after each COAM injection) were tested in an IFN-β ELISA (PBL Interferon Source). All samples were below the detection limit of 15.6 pg/ml, ruling out the possibility that the observed reduction in EAE with COAM in these mice is due to high IFN-β levels.

In a second set of experiments, we tested whether COAM could induce the production of IFN-β in primary SJL/J mouse embryonic cell cultures. Mixtures of fibroblasts, endothelial and epithelial cells were stimulated with different concentrations of COAM (1, 10, 30, 100, 300, 1,000 μg/ml) for 24, 48 or 72 hours, and IFN-β mRNA levels were measured in cell extracts using qPCR, revealing only marginal background levels of IFN-β mRNA and no differences between the different time points or concentrations of COAM (Table 2). Also a biological assay with L929 cells revealed no IFN-like activity in the supernatants of these cultures (<3.16 units/ml), despite detectable antiviral activity in samples containing 100 μg/ml or higher concentrations of COAM (titers between 1.0 to 1.5 log_10_ units/ml). This was equivalent to the effect of 1,000 μg/ml of COAM directly administered to the L929 cells and was thus explained as antiviral COAM carry-over on these test cells [18]. To test whether IFN-β induction took place at an earlier time point, mouse embryonic cell cultures were incubated with COAM (100, 1,000 μg/ml) for 4, 8 and 16 hours. Again, addition of COAM did not result in an increase of IFN-β mRNA copies (Table 2), nor in measurable titers of IFN-like bioactivity (<3.16 units/ml). Hence, COAM significantly diminished SCH-induced hyperacute EAE, but did not induce IFN. Therefore, alternative molecular and cellular mechanisms for the protective effects of COAM against EAE were examined.

**Table 2:** **COAM does not induce IFN-**β

**Incubation time**	**COAM (μg/ml)**						
	**1**	**10**	**30**	**100**	**300**	**1,000**	**control**
4 hours	ND	ND	ND	1.17 ± 0.20	ND	0.91 ± 0.31	1.00 ± 0.12
8 hours	ND	ND	ND	1.33 ± 0.35	ND	1.07 ± 0.11	1.00 ± 0.1
16 hours	ND	ND	ND	0.98 ± 0.18	ND	1.62 ± 0.46	1.00 ± 0.25
24 hours	1.12 ± 0.13	0.90 ± 0.15	0.19 ± 0.06	0.69 ± 0.37	0.17 ± 0.03	1.32 ± 0.65	1.00 ± 0.29
48 hours	1.11 ± 0.19	0.52 ± 0.09	1.92 ± 0.05	1.44 ± 0.25	1.55 ± 0.66	0.65 ± 0.39	1.00 ± 0.22
72 hours	1.28 ± 0.26	0.47 ± 0.14	1.43 ± 0.06	1.38 ± 0.38	1.5 ± 0.62	1.86 ± 0.59	1.00 ± 0.41

Primary SJL/J mouse embryonic cells were incubated with different concentrations of COAM (1 to 1,000 μg/ml) for various time intervals. After the indicated incubation period, cells were lysed and total cellular RNA was subjected to qPCR. IFN-β mRNA expression levels were measured and normalized against untreated controls. Data represent mean values of fold IFN-β mRNA level ± SEM of two independent experiments (n=2-4). COAM, chlorite-oxidized oxyamylose; ND = not determined; SEM, standard error of the mean.

### COAM reduces cell infiltration into the CNS

**Figure 3:**
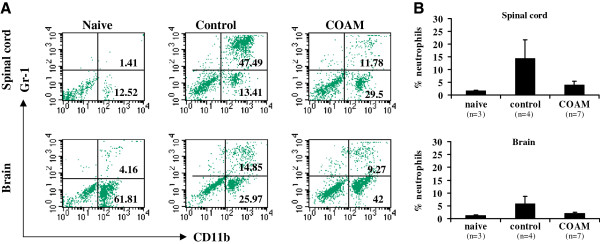
**Decreased numbers of neutrophils in the spinal cord and brain of COAM-treated mice.** (**A**) Flow cytometry analysis for the indicated markers was used to identify leukocytes isolated from the spinal cord and brain. In this experiment cells were isolated on day 16 after immunization and stained for CD11b and Gr-1. Numbers indicated on each graph represent the percentages of CD11b^+^Gr-1^+^ and CD11b^+^Gr-1^-^ cells. Each plot represents a staining pattern of pooled cells from three mice of one out of four representative experiments. (**B**) Morphologic examination of spinal cord and brain cytospin preparations stained with Hemacolor on day 16 after immunization after treatment with COAM. Bars represent averages ± SEM of at least three analyses; numbers of analyses are indicated between brackets; in each experiment cells from two mice were pooled per group. COAM, chlorite-oxidized oxyamylose; SEM, standard error of the mean.

**Figure 4:**
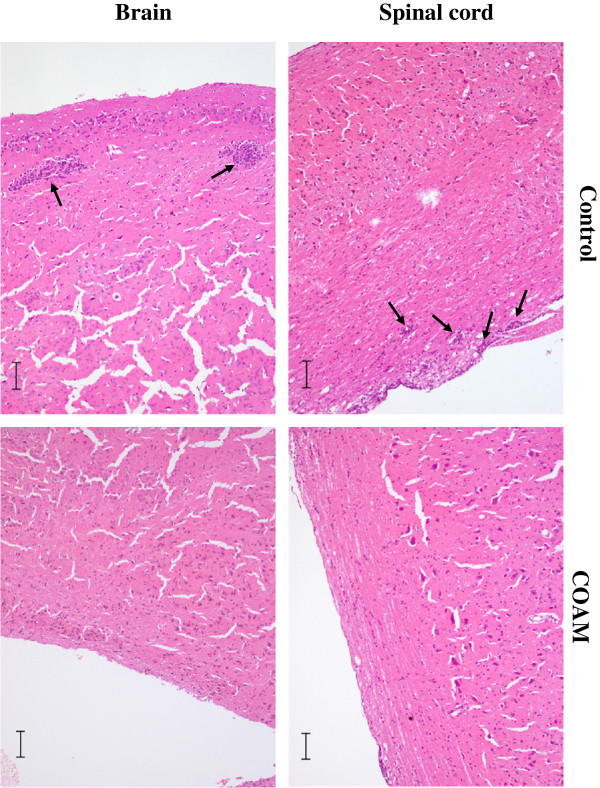
**Histological analysis of spinal cord and brain sections after treatment with EAE.** Spinal cords and brains from COAM- (n = 2) and saline-treated EAE animals (n = 2) were isolated during acute disease (day 17 after immunization) and fixed in 4% formol. Four micron thick paraffin sections were stained with H & E. Scale bars represent 100 μm. In the control sections, black arrows indicate prominent leukocyte infiltrations and at the surface perimeningeal leukocytes are visible. After treatment with COAM the numbers and sizes of these infiltrations were reduced. COAM, chlorite-oxidized oxyamylose; EAE, experimental autoimmune encephalomyelitis.

A prominent feature in chronic neuroinflammation is cell infiltration into the cerebrospinal fluid and CNS parenchyma. Infiltrates consist mainly of mononuclear cells (monocytes, dendritic cells, natural killer cells, CD4^+^ and CD8^+^ lymphocytes) [2]. In hyperacute EAE models, as the one used here, CNS neutrophils increase considerably [1]. To test whether inflammatory myeloid cell numbers were altered in the CNS of i.p. COAM-treated animals versus control saline-treated animals we isolated spinal cords and brain tissues during early and late stages of hyperacute EAE and analyzed these by flow cytometry. A cytometry staining pattern with indication of the percentages of CD11b^+^Gr-1^+^ cells, representative for data obtained in four experiments, is shown in Figure 3A. Following EAE induction, an increase in the number of neutrophils isolated from spinal cords and brains by day 16 after immunization was striking. We observed that neutrophil infiltration in the spinal cords and brains of COAM-treated mice was not different before onset of disease (day 5 and 9 after immunization, data not shown) compared with the control group receiving saline. During the acute phase of disease (day 16 after immunization; saline-treated mice selected with disease scores = 3.5, COAM-treated mice selected with disease scores = 4) a substantially smaller CD11b^+^Gr-1^+^ population was observed in the CNS of the COAM-treated group, as illustrated for one experiment (Figure 3B). When the results of four independent experiments were pooled, a lower percentage of CD11b^+^Gr-1^+^ cells in the spinal cord (21.76 ± 5.09%; *P* <0.05) of COAM-treated (mean disease score: 2.64) versus saline-treated animals (39.41 ± 5.03% and 16.75 ± 5.27% for spinal cord and brain respectively; mean disease score = 2.17) with symptoms of EAE (day 14 to 16) was observed. No overt differences in the populations of macrophages (CD11b^+^F4/80^+^ cells), CD4^+^ and CD8^+^ cells between both groups were detected (data not shown). By investigation of cytospin slides, we obtained data in accordance with the FACS results. Histological examination of longitudinal sections of the entire spinal cord and different brain sections obtained from mice with signs of EAE revealed that, in comparison with saline-treated mice, COAM reduced meningeal inflammation and numbers as well as sizes of perivascular infiltrates (Figure 4). These combined data demonstrated that COAM reduced absolute and relative numbers of trafficking neutrophils into the CNS (based on the flow cytometric data) and inhibited neuroinflammation and hyperacute EAE symptoms as seen by histopathology analysis and clinical evaluations.

### COAM alters expression of inflammatory chemokines and their receptors in the CNS of hyperacute EAE mice

**Figure 5:**
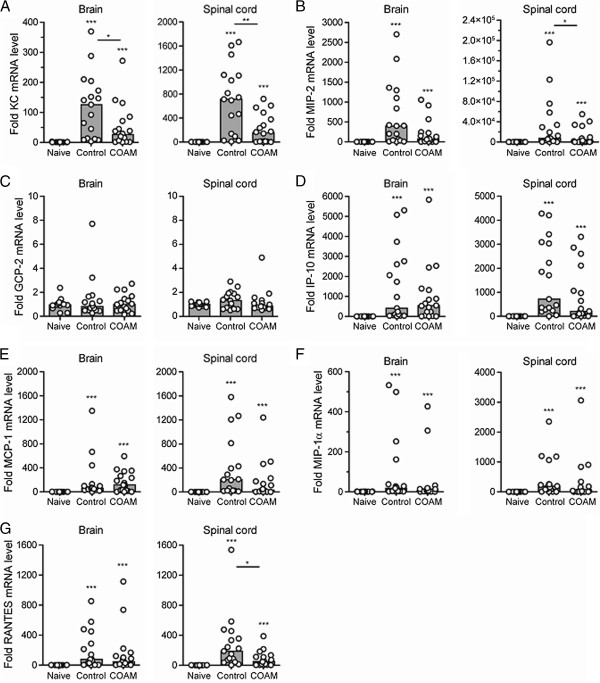
**Inflammatory chemokine expression in the CNS of hyperacute EAE control and COAM-treated mice.** Mice were injected i.p. with a dose of 2 mg of COAM on day 0 and 7 after induction of EAE. CNS cells were harvested on day 11 or 12 post immunization. Fold mRNA levels of the CXC chemokines (**A**) KC, (**B**) MIP-2, (**C**) GCP-2 and (**D**) IP-10, and of the CC chemokines (**E**) MCP-1, (**F**) MIP-1α and (**G**) RANTES were determined by qPCR. Histograms and dots indicate, respectively, group medians and range of individual data points (for each cohort the number of mice (n) exceeded 10, 13≤n≤18). Differences between COAM-treated EAE mice, untreated EAE controls and naive mice were statistically analyzed by the Mann Whitney test. Alterations in control hyperacute EAE mice or COAM-treated hyperacute EAE mice versus naive mice are indicated on top of every cohort. Pairwise significant comparisons between saline-treated control and COAM-treated hyperacute EAE mice are indicated by horizontal lines above the compared groups. * *P* <0.05; ** *P* <0.01; *** *P* <0.001. CNS, central nervous system; COAM, chlorite-oxidized oxyamylose; EAE, experimental autoimmune encephalomyelitis.

As COAM dampened clinical hyperacute EAE signs and local cellular signs of CNS neuroinflammation, we investigated the mRNA expression pattern of inflammatory chemokines in the CNS of naive, saline-treated and COAM-treated hyperacute EAE animals. At day 11 or 12 post immunization, that is, a time point when clinical disease became apparent in saline-treated hyperacute EAE mice, brains and spinal cords were analyzed for expression of the neutrophil chemokines KC/CXCL1, MIP-2/CXCL2 and GCP-2/CXCL6, of chemoattractants predominant for monocytes and activated T cells (MCP-1/CCL2, MIP-1α/CCL3 and RANTES/CCL5) as well as of IP-10/CXCL10, which is specific for activated T cells. In EAE-induced mice, both saline-treated as well as COAM-treated, mRNA levels for all these chemokines, except for GCP-2/CXCL6, were significantly upregulated in brains as well as in spinal cords, compared to levels in naive control mice (Figure 5). Compared to saline-treated EAE-induced animals, treatment with COAM resulted in significant decreases of KC, MIP-2 and RANTES in CNS tissues. Specifically, the brain content of mRNAs encoding KC decreased significantly after COAM treatment (decrease of median by 4.7 fold, *P* <0.05) and MIP-2 mRNA showed a trend towards decrease (*P* = 0.0774). In spinal cords, chemokine mRNA levels followed a decreasing course for IP-10 (*P* = 0.0535), MIP-1α and MCP-1. For the neutrophil chemokines KC and MIP-2, mRNA expression levels after COAM treatment were significantly decreased in spinal cords (decrease of median by 4.6 fold (*P* <0.01) and 17.7 fold (*P* <0.05), respectively), compared to saline-treated hyperacute EAE mice. The spinal cords from COAM-treated hyperacute EAE animals also exhibited significantly reduced expression levels of RANTES mRNA (decrease of median by 3.6 fold, *P* <0.05). The mRNA levels of GCP-2 remained unchanged in spinal cords and brains after treatment of hyperacute EAE mice with COAM. In conclusion, the expression of specific chemokines for neutrophils and other leukocyte types was decreased in the CNS by COAM.

**Figure 6:**
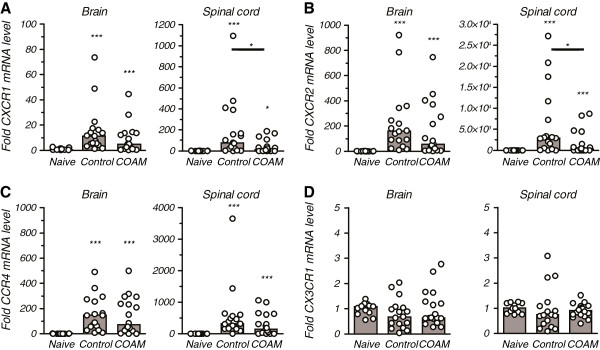
**Chemokine receptor expression in the CNS of hyperacute EAE control and COAM-treated mice.** SJL/J mice were immunized with SCH in CFA and were administered i.p. injections of COAM on days 0 and 7. Brain and spinal cord tissue were isolated on day 11 or 12 post immunization. mRNA expression of chemokine receptors (**A**) CXCR1, (**B**) CXCR2, (**C**) CCR4 and (**D**) CX3CR1 were analyzed by qPCR. Histograms and dots indicate, respectively, group medians and range of individual data points (for each cohort 13≤n≤18). * *P* <0.05; *** *P* <0.001 compared to naive mice as determined by the Mann Whitney test. Pairwise significant comparisons between saline-treated and COAM-treated hyperacute EAE mice are indicated by horizontal lines. CNS, central nervous system; COAM, chlorite-oxidized oxyamylose; EAE, experimental autoimmune encephalomyelitis; SCH, spinal cord homogenate.

In order to further characterize the CNS chemokine milieu associated with reduced disease symptoms in COAM-treated mice, we determined the expression levels of specific chemokine receptors by qPCR analysis. CXCR1 and CXCR2 are the most relevant chemokine receptors of neutrophils. CXCR2 is the receptor of MIP-2 and KC, whereas GCP-2 acts on both receptors CXCR1 and CXCR2. CCR4 and CX3CR1 play important roles in regulating the traffic of peripheral leukocytes to the inflamed CNS [27,28]. During onset of clinical disease (day 11 to 12 after immunization) CXCR1, CXCR2 and CCR4 mRNA levels in the brain and spinal cord were significantly increased compared to naive control mice (Figure 6). We detected significantly lower expression levels of CXCR1 and CXCR2 in COAM-treated mice than in saline controls. mRNA levels for CXCR1 and CXCR2 declined significantly in the spinal cords after the administration of COAM (decrease of median by 5.1 fold (*P* = 0.031) and 8.5 fold (*P* = 0.033), respectively), whereas the mRNA changes for CCR4 were not significantly different between COAM-treated and control mice. The mRNA levels of CX3CR1 remained unchanged in spinal cords and brains after treatment of hyperacute EAE mice with COAM. Together, these data showed that COAM downregulated the expression of chemokine receptors mediating chemotactic activity of neutrophils, but had no effect on the regulation of CNS CCR4 expression.

### Effects of COAM on blood cells and splenocytes

**Figure 7:**
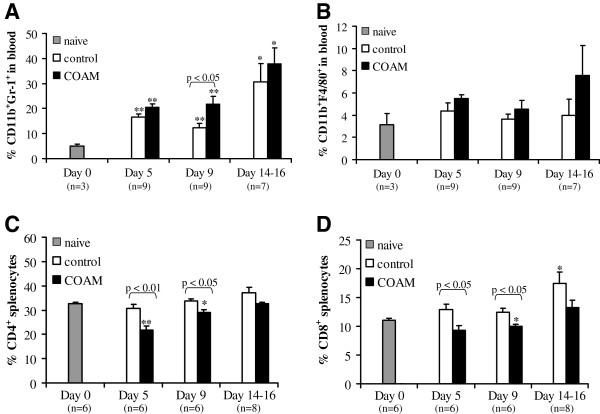
**Leukocytes in blood and spleen in response to COAM during hyperacute EAE.** Mice were immunized for hyperacute EAE and administered 2 mg COAM i.p. on days 0 and 7. Blood cells and splenocytes were collected on days 5, 9 and 14 to 16 after immunization. (**A**) Blood cells were stained with anti-CD11b-FITC and anti-Gr-1-PE and this analysis demonstrated COAM-induced transient blood neutrophilia at day nine in hyperacute EAE-induced mice. (**B**) Macrophages in the circulation were assessed by FACS analysis with anti-F4/80-PE and anti-CD11b-FITC. (**C**) COAM induced a decrease in the number of spleen CD4^+^ lymphocytes on days five and nine. (**D**) CD8^+^ splenocytes were identified with anti-CD8-FITC. (**A**-**D**) The data were combined from three independent experiments. Bars indicate the means ± SEM of three to nine mice. * *P* <0.05; ** *P* <0.01 for comparison with naive mice; Mann Whitney test. Pairwise comparisons between saline- and COAM-treated hyperacute EAE mice are indicated above the histograms. COAM, chlorite-oxidized oxyamylose; EAE, experimental autoimmune encephalomyelitis; FITC, fluorescein isothiocyanate; PE, phycoerythrin; SEM, standard error of the mean.

To investigate whether COAM caused changes in leukocyte numbers in anatomical compartments other than the CNS, blood cells and splenocytes were isolated on days 5, 9 and 14 to 16 after immunization and analyzed by flow cytometry. Immunization for hyperacute EAE induced a significant increase in the number of neutrophils (ranging between 15% to 30%) in the circulation compared to non-immunized mice (5%), in accordance with previous observations [29,30]. In mice treated i.p. with COAM, higher percentages of neutrophils were observed in the circulation than in naive mice, with only a significant difference between saline and COAM at day nine after immunization (*P* <0.05) (Figure 7A). No significant differences in the percentages of circulating monocytes were observed between naive, saline-treated and COAM-treated EAE animals (Figure 7B). Spleen cells of immunized COAM-treated and saline-treated mice contained about equal percentages of neutrophils and macrophages (data not shown), whereas a decrease in the number of CD4^+^ (Figure 7C) and CD8^+^ (Figure 7D) splenocytes was observed after treatment with COAM, with significant differences at days five and nine after immunization.

### COAM drastically alters leukocyte populations at the injection site

**Figure 8:**
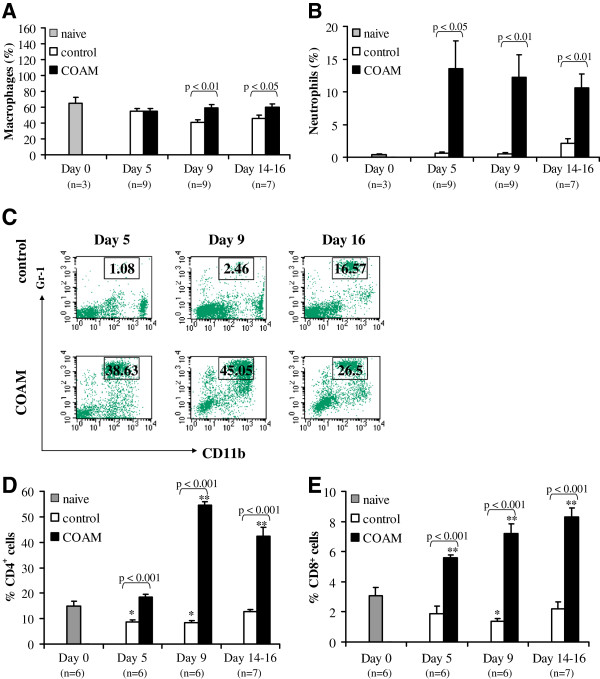
**COAM induces peritoneal alterations in leukocyte populations in animals with hyperacute EAE.** Peritoneal cells were collected on days 0, 5, 9 and 14, 15 or 16 post immunization with SCH in SJL/J mice. (**A**) The percentages (morphologic examinations) of peritoneal macrophages and (**B**) neutrophils in the peritoneal exudates were compared between naive mice and EAE-induced mice, treated i.p. with saline or with COAM. Data are compiled from three experiments; bars represent averages ± SEM of three to nine mice. Statistical significance was determined using the Mann Whitney test. (**C-E**) Flow cytometric analysis of leukocytes in the peritoneal cavity of COAM-treated versus saline-treated EAE mice. Cells were stained for the presence of CD11b, Gr-1, CD4 and CD8. (**C**) Example of FACS analysis with indication of gating of double positive cells. The numbers within the gates indicate the percentages of Gr-1-positive cells present in the whole sorted peritoneal lavages. One representative analysis out of nine is shown. (**D-E**) The data of three experiments were pooled and the percentages of CD4^+^ and CD8^+^ cells on each of the examined days are shown. Bars represent averages ± SEM, the individual numbers of animals (n) for each condition are indicated below each of the histograms. Asterisks indicate * *P* <0.05; ** *P* <0.01 for comparison with naive mice (Mann Whitney test). Pairwise comparisons (*P* values) between saline- (control) and COAM-treated hyperacute EAE mice are indicated on top of the histograms. COAM, chlorite-oxidized oxyamylose; EAE, experimental autoimmune encephalomyelitis; SCH, spinal cord homogenate; SEM, standard error of the mean.

Finally, the effects of COAM on leukocyte populations at the injection site were studied. Mice were induced for hyperacute EAE, treated i.p. with 2 mg COAM on days 0 and 7 after immunization with SCH and sacrificed at different time points after immunization. Peritoneal lavage fluids were collected for morphologic examination on day 5, that is, the preclinical phase; day 9, that is, shortly before the expected onset of clinical symptoms of hyperacute EAE; and on days 14, 15 or 16, which corresponded with the peak of disease. Diseased mice were selected so that their mean clinical scores were comparable between COAM- and saline-treated groups. Peritoneal cells were cytospun onto slides, stained with Hemacolor, and leukocyte subsets were counted. Naive and saline-treated hyperacute EAE mice on day 5 after immunization yielded 1.47 to 1.93 x 10^6^ cells/ml, comprising high numbers of macrophages (65.13% and 54.89% of total cells, respectively) and a small proportion of neutrophils (0.4% and 0.58% for naive and saline-treated hyperacute EAE mice, respectively). COAM induced a peritoneal leukocyte influx nine days after EAE induction (*P* <0.05 versus saline control). Macrophages (Figure 8A) and particularly neutrophils (Figure 8B) were found to be significantly increased in COAM-treated mice. These data are in line with previous findings in a virus infection model [19]. These effects of COAM were detectable in hyperacute EAE animals even on day 14, one week after the last COAM injection on day 7. FACS analyses reinforced these cytospin data. The peritoneal fluid of COAM-treated mice contained an excessive proportion of CD11b^+^Gr-1^+^ cells compared with saline-treated mice on each of the examined days (Figure 8C). When the numbers of CD11b^+^Gr-1^+^ cells from three independent experiments were expressed as percentages of total cell numbers collected per mouse, peritoneal fluids of hyperacute EAE-induced COAM-treated mice were shown to contain approximately four times higher numbers of neutrophils than those of saline-treated hyperacute EAE mice. For the analysis of defined lymphocyte subtypes in animals treated with COAM, peritoneal fluid cells were stained with anti-CD4-PE and anti-CD8-FITC antibodies. COAM injection in immunized mice significantly increased the percentage of peritoneal CD4^+^ and CD8^+^ cell numbers as compared to saline-treated EAE mice (Figure 8D and E). With the use of *in vivo* imaging, we could demonstrate that i.p. injected labeled COAM remained mainly in the peritoneal cavity for prolonged time intervals, whereas it disappeared quickly from the mice after intravenous injection (data not shown).

**Figure 9:**
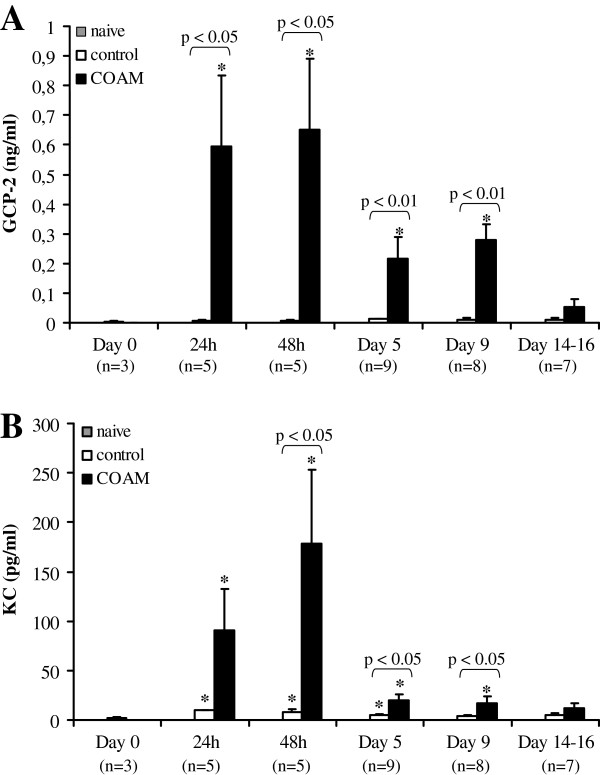
**GCP-2 and KC are upregulated in the peritoneum in response to COAM.** SJL/J mice were induced for EAE by injection with SCH and treated with COAM (2 mg i.p., day 0 and 7 post immunization) or a control solution of saline. At different time points after COAM injection, peritoneal fluid was collected by i.p. lavage. Protein levels of GCP-2 and KC in peritoneal fluids were detected by ELISA. The total number (n) of mice examined per group are indicated between brackets; histograms indicate group means ± SEM. Compared to the naive levels, significant *P*-values of (*) *P* <0.05 were obtained (Mann–Whitney test). Pairwise comparisons between saline- and COAM-treated hyperacute EAE mice are indicated above the histograms. GCP-2 and KC levels in all naive mice (without EAE induction) were below the detection limit. COAM, chlorite-oxidized oxyamylose; EAE, experimental autoimmune encephalomyelitis; SCH, spinal cord homogenate; SEM, standard error of the mean.

Mouse CXC chemokines that are known to specifically attract neutrophils include GCP-2, KC and MIP-2 [31]. We studied whether these chemokines are induced in the peritoneum of EAE-immunized mice in response to COAM. Indeed, GCP-2 was present in the peritoneal lavage fluid 24 hours after the first COAM injection, whereas GCP-2 was barely detectable in saline-treated control mice (Figure 9A). Induction of EAE caused an upregulation of KC expression (Figure 9B) and protein levels of GCP-2 and KC were found to be significantly increased over saline controls at 24 hours, 48 hours, day 5 and day 9 after COAM administration. Levels of MIP-2 expression and of the cytokine IL-17 were below the detection limits. Hence, induction of GCP-2 and KC by COAM may constitute a chemotactic force for mobilization of neutrophils, and this favors the proposal of retention of immune cells at the COAM-injection site, rather than of increased cell proliferation.

In conclusion, COAM induced significant leukocyte recruitment at the peripheral injection site. Specifically, neutrophils as fast contributors to hyperacute neuroinflammation and also monocytes and lymphocytes as slower effector cells in acute inflammatory reactions were recruited to the periphery by COAM injection.

## Discussion

The present study provides a novel approach with therapeutic potential for the treatment of hyperacute CNS inflammation, namely by deviation of leukocyte trafficking from the CNS to peripheral body compartments. Furthermore, we provide an example of a pharmacological agent to attain this goal *in vivo*. Two rationales prompted us to evaluate COAM in EAE: (i) the suggestion [16] that COAM induces endogenous IFN-β with known therapeutic effects in MS and EAE and (ii) the fact that COAM protected against immunopathologies observed in acute lethal virus infection models [17,19]. Our data provide no evidence for the first possibility and stimulated research into alternative explanations of mechanisms of action. Indeed, by a number of experimental approaches, including IFN bioassays, ELISAs and mRNA titrations in hyperacute EAE animals and on MEF cell cultures, we corroborated and extended recent data that COAM does not induce IFN-β *in vitro* and *in vivo*, but instead affects the chemokine system. Our present data are in line with findings in infection and tumor animal models [18,19,32].

One effect of COAM in the CNS is the reduction of KC/CXCL1 expression in brain and spinal cord and of MIP-2/CXCL2 and RANTES/CCL5 expression in the spinal cord. Reduction of specific chemokine expression levels may contribute to the observed significantly reduced leukocyte counts in the CNS. After treatment with COAM, reduced neuroinflammation was paralleled by significant reduction in hyperacute EAE disease scores. Further analyses were done to determine how COAM may affect trafficking of inflammatory cells. COAM treatment increased circulating and peritoneal neutrophil counts, in line with less influx into the CNS and compartmental deviation of neutrophils out of the CNS towards the COAM injection site. The latter was also observed in another study [19], in which it was found that i.p. COAM bound GCP-2/CXCL6 leading to enhanced chemotaxis of myeloid cells into the peritoneal cavity. The present study thus provides a practical example for the thesis that modulation of the chemokine system may be exploited therapeutically to reduce leukocyte infiltration into the CNS. Blocking of chemokine activity by binding to glycosaminoglycans (GAGs) was proposed as an anti-inflammatory modus [33,34]. However, with COAM a different action of GAG mimicry is proposed: one that does not block but instead enhances chemokine activities in specific body compartments. Collectively, our findings lead to the suggestion that at the injection site COAM captures endogenous chemokines from the environment and creates a potent chemotactic gradient and thus lures inflammatory cells from the whole body into a specific compartment. Furthermore, these findings are complemented by the fact that COAM reduces CNS expression of specific chemokines with significant reduction of leukocytes in the CNS. We coined the term ‘cell deviation therapy’ for this concept.

Another way to decrease CNS infiltration by leukocytes is with the adhesion inhibitor Natalizumab/Tysabri, but this treatment increases the risk of polyomavirus infections [35-37]. COAM, being an immunomodulating agent with potent antiviral activity, might be an alternative treatment option for patients infected with such viruses, but this remains to be proven. Finally, the combination of COAM and Tysabri with complementary modes of therapeutic and antiviral actions constitutes another future approach to combat various forms of neuroinflammation.

## Conclusions

Treatment of SCH-induced hyperacute neutrophilic EAE in SJL/J mice and of MOG-peptide-induced EAE in IFN-γ-deficient BALB/c mice with COAM resulted in significant amelioration of disease symptoms. This study provides a novel mechanism and an interesting molecular probe to study leukocyte deviation. Our data might have implications for the treatment of inflammatory and autoimmune diseases such as EAE and MS.

## Abbreviations

BBB: Blood–brain barrier; CFA: Complete Freund’s adjuvant; CNS: Central nervous system; COAM: Chlorite-oxidized oxyamylose; EAE: Experimental autoimmune encephalomyelitis; ELISA: Enzyme-linked immunosorbent assay; FCS: Fetal calf serum; H & E: Haematoxylin and eosin; IFA: Incomplete Freund’s adjuvant; IFN: Interferon; i.p.: Intraperitoneally; i.v.: Intravenously; mAb: Monoclonal antibody; MEF: Mouse embryonic fibroblasts; MEM: Modified Eagle’s medium; MOG: Myelin oligodendrocyte glycoprotein; MS: Multiple sclerosis; PBS: Phosphate-buffered saline; SCH: Spinal cord homogenate; qPCR: Quantitative polymerase chain reaction.

## Competing interests

GO and JVD are inventors of intellectual property from the present study, which is protected and owned by Leuven Research and Development (LRD) for the University of Leuven. All other authors declare they have no competing interests.

## Authors’ contributions

NB made substantial contributions to the conception and design, acquisition of data, analysis and interpretation of results and drafting of the manuscript and part of these data formed the basis of her doctoral dissertation. HH made substantial contributions to the conception and design, acquisition of data, interpretation of results and drafting the manuscript and was PhD thesis promoter of NB. SL contributed experimental help with the qRT-PCR and design, analysis and interpretation of data. EM helped with the acquisition of data and interpretation. PM provided the IFN-γ-deficient mice and critically analyzed the data. LS made substantial contributions to the interpretation of data. JVD made substantial contributions to the conception and design, analysis and interpretation of results and promoted the COAM project. GO made substantial contributions to the conception and design, interpretation of results, drafting the manuscript, has given final approval of the version to be published and was promoter of the doctoral thesis of SL and of the COAM project. All authors critically read and approved the final manuscript.
